# Localization of myocardial scar in patients with cardiomyopathy and left bundle branch block using electrocardiographic Selvester QRS scoring - comparison with cardiac magnetic resonance

**DOI:** 10.1186/1532-429X-15-S1-P61

**Published:** 2013-01-30

**Authors:** Bjorn Wieslander, Katherine Wu, Zak Loring, Linus Andersson, Terry Frank, Gary Gerstenblith, Gordon F Tomaselli, Robert G Weiss, Galen S Wagner, Martin Ugander, David G Strauss

**Affiliations:** 1 Clinical physiology, Karolinska Institutet and Karolinska University Hospital, Stockholm, Sweden; 2 Division of cardiology, department of Medicine, Johns Hopkins Medical Institutions, Baltimore, MD, USA; 3 School of Medicine, Duke University, Durham, NC, USA; 4 Duke Clinical Research Institute, Duke University, Durham, NC, USA; 5 Office of Science and Engineering Laboratories, Center for Devices and Radiological Health, United States Food and Drug Administration, Silver Spring, MD, USA

## Background

The left ventricular (LV) lead in cardiac resynchronization therapy (CRT) can be placed on any non-septal LV wall, most commonly the lateral wall. Presence of myocardial scar adversely affects CRT outcome, particularly if it involves the LV pacing site. It is increasingly important to identify and localize myocardial scar in CRT candidates, the majority of whom have left bundle branch block (LBBB). We aimed to describe the diagnostic performance of electrocardiographic (ECG) criteria based on the Selvester QRS score in localizing myocardial scar into 5 LV wall segments and in detecting non-septal scar in patients with LBBB using Cardiac Magnetic Resonance as the gold standard.

## Methods

In 39 cardiomyopathy patients with strictly defined LBBB (17 with scar, 22 without scar), late gadolinium enhancement cardiac magnetic resonance images (CMR-LGE) and 12-lead ECGs were analyzed for scar presence in 5 LV wall segments. Using stepwise regression analysis, multiple subsets of the Selvester QRS score ECG criteria were identified for each of the 5 individual segments and for the 4 non-septal segments together. The diagnostic performance of each of these subsets is displayed in the graph below. The one subset for each location that had the highest combination of sensitivity plus specificity was selected.

## Results

The best identified subset of Selvester QRS score criteria for screening of non-septal scar had 75% (95% CI: 51-90%) sensitivity, 95% (78-99%) specificity, 92% (67-99%) positive predictive value and 84% (65-94%) negative predictive value. For each individual wall segment, 40-60% sensitivities and 77-100% specificities were found.

## Conclusions

The 12-lead ECG can convey information about scar presence and location in patients with cardiomyopathy and LBBB. ECG screening criteria for scar in potential CRT LV pacing sites were identified. Further exploration is required to determine the clinical utility of the 12-lead ECG in combination with other imaging modalities to screen for scar in potential LV pacing sites in CRT candidates.

## Funding

The study was supported by the National Heart, Lung, and Blood Institute, National Institutes of Health (HL103812 to KCW, HL91062 to GFT, and HL61912 to RGW), the DW Reynolds Foundation and the FDA Critical Path Initiative.

**Figure 1:**
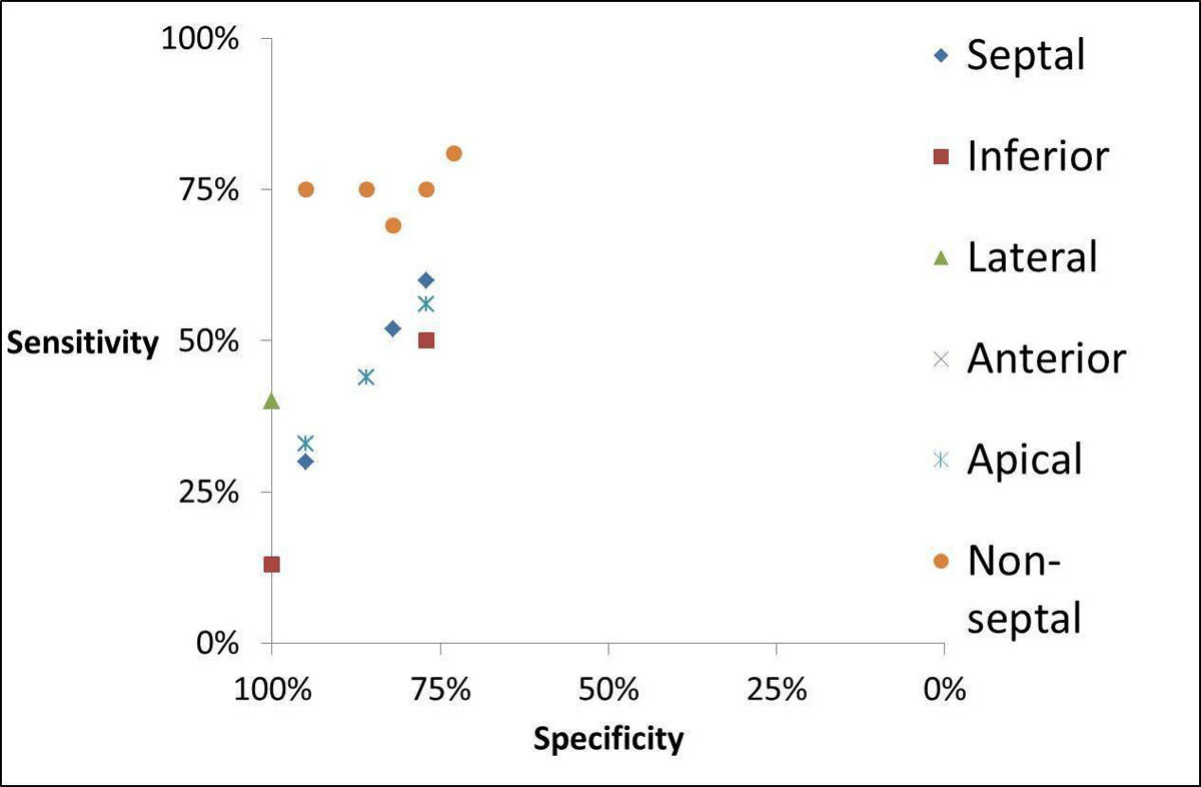
This sensitivity vs 1-specificity graph displays the diagnostic performances of all the subsets of the Selvester QRS score ECG criteria that were identified in detecting myocardial scar in each of the five LV wall segments, as well as in the four non-septal segments together.

